# In vitro anti-influenza assessment of anionic compounds ascorbate, acetate and citrate

**DOI:** 10.1186/s12985-022-01823-0

**Published:** 2022-05-23

**Authors:** Hadiseh Shokouhi Targhi, Parvaneh Mehrbod, Fatemeh Fotouhi, Mehriar Amininasab

**Affiliations:** 1 Department of Cell and Molecular Biology, Kish International Campus, University of Tehran, Kish Island, Iran; 2 Influenza and Respiratory Viruses Department, Pasteur Institute of Iran, Tehran, Iran; 3 Department of Cell and Molecular Biology, School of Biology, College of Science, University of Tehran, Tehran, Iran

**Keywords:** Ascorbate, Acetate, Citrate, Cytokine, Influenza A virus

## Abstract

**Background:**

Influenza A virus (IAV) infection remains a serious public health threat. Due to drug resistance and side effects of the conventional antiviral drugs, repurposing the available natural compounds with high tolerability and fewer side effects has attracted researchers’ attention. The aim of this study was to screen in vitro anti-influenza activity of three anionic compounds ascorbate, acetate, and citrate.

**Methods:**

The non-cytotoxic concentration of the compounds was determined by MTT assay and examined for the activity against IAV in simultaneous, pre-, and post-penetration combination treatments over 1 h incubation on Madin-Darby Canine Kidney (MDCK) cell line. The virus titer and viral load were determined using hemagglutination assay (HA) and qPCR, respectively. Few pro-inflammatory and anti-inflammatory cytokines were evaluated at RNA and protein levels by qPCR and ELISA, respectively.

**Results:**

The non-cytotoxic concentrations of the ascorbate (200 mg/ml), acetate and citrate (both 3 mg/ml) reduced the viral titer by 6.5, 4.5, and 1.5 logs in the simultaneous combination treatment. The M protein gene copy number decreased significantly in simultaneous treatment (*P* < 0.01). The expression of cytokines was also affected by the treatment of these compounds.

**Conclusions:**

These anionic compounds could affect the influenza virus load, thereby reducing pro-inflammatory cytokines and increasing anti-inflammatory cytokines levels.

**Supplementary Information:**

The online version contains supplementary material available at 10.1186/s12985-022-01823-0.

## Background

Influenza A virus (IAV) from *Orthomyxoviridae* family with eight segments of negative-sense single-stranded RNA genome is one of the most common pathogens with morbidity and sometimes high mortality worldwide [1]. It has caused severe respiratory disease for hundreds of years in humans and different animal species [2] and causes over 50,000 people decease each year during seasonal influenza virus infection [3]. According to CDC report in 2009, the global spread of 2009 pandemic of H1N1 (pH1N1) influenza virus, which resulted from the reassortment of the eight distinct genome RNA segments of two strains of influenza virus within a single host cell, infected over 22 million people [3].

Humans are limited in their ability to control influenza infection [4] and it seems the vaccination programs are the best approach for preventing influenza pandemics in the future. On the other hand, the interval between characterization of the new strain and vaccine production might be disastrous [4, 5]. It is critical to act quickly to develop new vaccines and highly effective antiviral agents for the treatment of all strains, subtypes, and pandemic influenza infections [5, 6].

The conventional drugs are neuraminidase inhibitors and ion-channel blockers. Oseltamivir and zanamivir as neuraminidase inhibitors are antiviral drugs that arrest neuraminidase to prevent releasing virus particles. They are used for both treatment and prophylaxis of influenza. Amantadine and rimantadine are matrix protein (M2) ion-channel inhibitors that block viral uncoating within host cells and have been used to treat flu infection [7–9]. Furthermore, antiviral resistance during therapy might have negative consequences for the central nervous system and the gastrointestinal tract, and has been disappointing [10]. All of these medicines have a vague clinical efficacy [11], and several incidences of drug resistance have been described [7].

Low-cost generic drugs easily accessible during pandemics should be considered as alternative treatments. This type of medication should target the host's immune response to lessen the consequences of influenza, by targeting necessary cellular proteins for influenza virus replication [5, 12, 13].

IAVs rely on the host cell machinery for the replication and transportation inside the cell [14]. New antiviral drugs should target overlapping pathways used by both host cells and influenza viruses. The benefit of this method involves reducing the risk of drug resistance [14, 15]. Previous researches have demonstrated that influenza infections cause uncontrolled increases in pro-inflammatory cytokines, making it as one of the major risk factors for serious consequences of flu infection [16, 17]. Tumor necrosis factor-α (TNF-α), interleukin-6 (IL-6) and interferon-γ (IFN-γ) are the most important cytokines involved in inflammation [18, 19] and could induce an innate immune system to manage the infection. Cytokines are necessary for a successful antiviral response; however, dysregulation of cytokines (hypercytokinemia) can cause potentially fatal responses [20]. Anti-inflammatory and immunomodulatory factors can be used as effective alternatives to vaccination and conventional antiviral medications [6].

There are two types of acids that can be utilized in disinfection methods: organic acids (e.g. citric, glutaric, lactic, mallic and propionic acids) and inorganic acids (e.g. nitric, hydrochloric, sulphuric and phosphoric acids). Both are effective against viruses that are sensitive to low pHs [21].

A previous study provided the rationale for using zinc **acetate**/carrageenan gels and their ability to block the sexual transmission of HIV and herpes simplex virus 2 ( HSV-2) [22]. Acetate protected against respiratory syncytial virus (RSV)-induced illness by modifying type 1 interferon responses and increment in interferon-stimulated gene expression in the lung epithelial cells, through a mechanism that involves activation of the membrane receptor GPR43. This data also supported the significance of using acetate as cheap interpositions for treating bronchiolitis caused by RSV [23].

A study displayed that an iron salt ferric ammonium citrate (FAC) prevented the infection of IAV, human immunodeficiency virus (HIV), Zika virus (ZIKV), and Enterovirus 71 (EV71). Mechanistically, FAC inhibited viral infection by inducing viral fusion and blocking endosomal viral release [24]. Vitamin C, ascorbic acid (AA), is a strong antioxidant that is needed for good health and immunological potentiation. For many years, it has been studied as a potential treatment for usual colds [25, 26]. Since ascorbic acid can apparently improve the response to stress in rat and influence the repair of skin, it might assist to repair the mucosal damage produced by viruses [27]. AA has become very popular for its antioxidant properties. It is considered as an essential co-substrate for a big class of enzymes and regulates gene expression by interacting with critical transcription factors. AA is crucial in all demanding conditions such as immune-stimulating, anti-inflammatory, antiviral and antibacterial functions, which all are related to inflammatory procedures [28]. Another study represented that the nutrient combination containing lysine, proline, ascorbic acid, green tea extract, N-acetyl cysteine (NAC), selenium, and different micronutrients is an effective inhibitor of influenza A virus growth as examined in MDCK and Vero cell cultures [29]. Also, vitamin C has been used to treat hepatitis, encephalitis, influenza, SARS, and a few different viral sicknesses [30].

In this study, the anti-influenza activity of anionic compounds ascorbate, acetate, and citrate was evaluated against influenza virus A/Puerto Rico/8/34 (H1N1) replication in vitro and accompanying anti-inflammatory responses.

## Methods

### Chemical compounds preparation

All proposed anionic compounds including ascorbic acid (CAS: 50-81-7), sodium acetate (CAS: 127-09-3) and sodium citrate (CAS: 6132-04-3) were supplied from Merck, Germany. Amantadine hydrochloride (CAS: 665-66-7) and oseltamivir phosphate (CAS: 204255-11-8) as antiviral control drugs were purchased from Sigma (St. Louis, MO, USA). The compounds were prepared by dissolving the powders in dH_2_O. The concentration of 200 mg/ml of sodium citrate and sodium acetate, and 300 mg/ml of ascorbic acid, 2 mg/ml of amantadine hydrochloride and 4 mg/ml of oseltamivir phosphate were prepared in Dulbecco’s Modified Eagle’s Medium (DMED) (Gibco, USA). Then, twofold serial dilutions of each compound were prepared from the initial prepared concentration.

### Cell culture and influenza virus propagation

Madin-Darby Canine Kidney (MDCK) cell line and influenza A virus PR/8/34 (H1N1) were obtained from Pasteur Institute of Iran, Influenza and Respiratory Viruses Department. MDCK cells were grown in Dulbecco’s Modified Eagle’s Medium (DMEM) (Gibco USA), containing 10% Fetal Bovine Serum (FBS) (Gibco USA) and 1% Penicillin/Streptomycin (Gibco USA) as antibiotic. Cells were incubated at 37 °C in a humidified 5% CO_2_ incubator. The virus was propagated in MDCK cells in the presence of 1 µg/ml of trypsin-Tosylamide Phenylethyl Chloromethyl Keton-treated Trypsin (TPCK) (Sigma, USA). After 48 h incubation the supernatant containing virus was harvested and titrated.

### Virus titration

In order to determine the virus titration, standard 50% tissue culture infectious doses (TCID_50_) method was used [31]. The cell culture medium was aspirated from 90% confluent 96-well plates seeded with MDCK cell and washed twice with phosphate-buffered saline (PBS). Then, 100 μl of tenfold dilutions of virus in DMEM was added into the wells. After 1 h, the supernatants were replaced with medium containing 1 μg/mL Trypsin-TPCK, and incubated at 37 °C for 48 h. Then, the cytopathic effect (CPE) was evaluated microscopically. Hemagglutination assessment was conducted to measure virus infectivity using Karber formula. The value 100 TCID_50_ of the virus was used for the antiviral assays [32, 33].

### Cytotoxicity of the compounds

The cytotoxic effects of the anionic compounds on the viability of MDCK cells was measured by MTT [3-(4, 5-dimethylthiazol-2ol) 2, 5 diphenyl tetrazolium bromide] assay. Briefly, the confluent 96-well plate of MDCK cells were exposed to twofold serial dilutions of the compounds (in triplicates), 100 μl/well, and incubated for 48 h. After the incubation period, the supernatants were removed and 100 μl of 1X MTT solution (100 μl/well) was added to each well. The plates were incubated for 4 h at 37 °C. Then, 100 μl of DMSO (Samchun, Korea) was added to dissolve formazan crystals. Color adsorption in the solution was measured using an enzyme-linked immunosorbent assay (ELISA) reader (StataFax 2100, USA) at 570 nm to calculate the 50% cytotoxic concentration (CC_50_). The cells without treatment were used as negative control. The percentage of toxicity was calculated using the following formula:

Toxicity (%) = [100 − (ODT/ODC) × 100], where ODT and ODC refer to the absorbance of the test substance and control, respectively [34]. The 50% cytotoxic concentration (CC_50_) was defined as the cytotoxic concentration of compounds by regression analysis.

The non-cytotoxic concentration (NCTC) of the compounds was also determined using the same procedure and used for the antiviral assays. Amantadine hydrochloride and oseltamivir phosphate were evaluated in the same way. The cells with no treatment were considered as negative control.

### Selectivity index

The selectivity index (SI) is relative safety of a compound and it was obtained by dividing CC_50_ by NCTC in the same units. Selectivity index value higher than 3 indicates potentially safe antiviral activity of a compound [35].

### Antiviral activity of the compounds

MDCK cells were exposed to three different combinations of virus and compounds (co-, pre- and post-penetration treatments). The cells were then washed and TPCK-containing medium (0.5 μg/ml) was added to each well (100 μl/well).

Amantadine hydrochloride and oseltamivir carboxylate were tested in parallel as control antiviral drugs. The cells with media only served as negative control. Following 48 h incubation at 37 °C, viabilities of the cells were evaluated by MTT assay as described above. Concurrently, the cell supernatants were exposed to hemagglutination assay (HA) for determining virus titers [36]. To confirm the antiviral results of HA assay, the samples of co-penetration treatments were quantified by TCID_50_ evaluation. The assay was performed on tenfold dilutions of the samples exposed to the cells for 48 h. Then, Karber formula was used to calculate the TCID_50_ results [32].

### Molecular assay

#### One-step quantitative real-time PCR for IAV detection

Viral RNA was extracted from the supernatants of 48 h treatments using High Pure Viral RNA Kit, according to the manufacturer’s instructions (Roche, Switzerland). The extracted RNAs were re-suspended in Elution Buffer and stored at -80 °C for one-step real-time PCR assay. The primers and probes were designed and synthesized by SinaClon Co. (Iran) based on the latest WHO guideline. The reaction mixture consisted of 2X Master Mix, SuperScriptIII RT/ Platinum Taq Mix, specific primers and fluorescent probes. The matrix gene primers and probe sequences, and the reactions cycling were used as mentioned before [37].

#### Quantitative real-time PCR for cytokines genes detection

Primers for the target genes were designed and synthesized by First Base Co., Malaysia and Inqaba Biotech, South Africa, respectively. The target genes consisted of five cytokine (i.e. TNF-α, IL-6, IL-27, IFN-β1 and CCL2) and two housekeeping genes (GusB and ActB) as described in our previous studies [36, 38].

MDCK cells were exposed to the treatment of compounds and influenza virus as described above. After 72 h infection period, the supernatants were harvested. High Pure RNA Isolation Kit was then used to extract the total RNA from supernatants according to the kit instruction (Roche, Germany). The isolated RNA samples were dissolved in RNAse-free distilled water and stored at − 80 °C.

The isolated RNA samples were subjected to cDNA synthesis using Transcriptor First Strand cDNA Synthesis kit (Roche, Germany). The reaction was carried out with 5X Transcriptor Reverse Transcriptase Reaction buffer, Random Hexamer primers, Protector RNase Inhibitor, dNTP mix and Transcriptor Reverse Transcriptase in a final volume of 20 μl. For primer annealing the mix was incubated at 25 °C for 10 min followed by 55 °C, 30 min duration, for reverse transcription and inactivated at 85 °C for 5 min. The synthesized cDNAs were stored at -20 °C for further usage. Virus-inoculated and mock-infected supernatants were used as positive and negative controls, respectively. The concentration of the cDNA templates were measured using a Picodrop Spectrophotometer system (Alpha, Biotech, UK).

Quantitative real-time PCR was carried out using different primers to evaluate the expression of selected genes. The reaction was performed by a Rotor-Gene 3000 (Corbett, Qiagen, Germany). PCR mixture contained 3 μl of synthesized cDNA solution, 5 μl 2× SYBR Green Master Mix (Thermo scientific, Lithuania) and 500 nM of each primer at a total volume of 20 μl. The amplification program started with a denaturation at 95 °C for 10 min followed by 45 cycles in which each cycle consisted of three steps, template denaturation at 95 °C for 15 s, primer annealing at 54–58 °C (for different genes) for 20 s, and primer extension at 72 °C for 25 s. All the PCR reactions were performed in duplicate. Following the amplification, melting curve analysis was performed to confirm the specificity of amplified products.

### Cytokine quantification

MDCK cells were treated as mentioned above. Untreated cells were considered as the negative control. Cell-free supernatant was harvested following 72 h infection period and stored at − 80 °C for the cytokine analysis. In order to investigate the effect of viral infection and anionic compounds on the production of inflammatory cytokines such as IL-6, CCL2 and TNF-α as well as anti-inflammatory cytokine proteins IFN-β1 and IL-27, ELISA method was utilized. Quantitative sandwich Picokine ELISA kits (Boster Biological Technology, CA, USA) were used to detect IL-6, TNF-α and IL-27 cytokine proteins according to the manufacturer's instructions. The CCL2 and IFN-β were evaluated by sandwich Ready-SET-Go kit (Invitrogen, USA) and sandwich geneILNB1 kit (EIAab Science Co, China) according to the manufacturers’ instructions, respectively. The concentrations of the cytokines were calculated according to the corresponding reaction standard formula.

### Statistical analysis

Data were presented as mean ± SD and analyzed by one-way analysis of variance (ANOVA) and General Linear Model (GLM) (SPSS 20.0) using Tukey multi-range post-hoc test to find differences between the experimental groups. Sample values with *P* ≤ 0.05 and *P* ≤ 0.01 were considered statistically significant and highly significant, respectively.

## Results

### Cytotoxicity and selectivity index of the compounds

The cytotoxicity of compounds was obtained to determine the non-cyotoxic concentrations prior to antiviral assay. The CC_50_ was obtained for each compound. The NCTC of the compounds were calculated from the MTT results by one-way ANOVA analysis and compared to negative control with no significant effect on the cell viability. The calculated selectivity index values of compounds were 1.43, 9.65 and 6.98 for ascorbate, citrate and acetate, respectively (Table 1).

**Table 1:** CC_50_, NCTC and selectivity index of the compounds

Compound	Quantity used (mg/ml)	CC_50_ (mg/ml)	NCTC (mg/ml)	Selectivity index (SI = CC_50_/NCTC)
Ascorbate	300	287 ± 1.69	200 ± 0.00	1.43
Acetate	200	28.95 ± 4.32	3.00 ± 0.00	9.65
Citrate	200	20.95 ± 10.64	3.00 ± 0.00	6.98
Amantadine hydrochloride	2	0.20 ± 1.53	0.10 ± 0.00	2
Oseltamivir carboxylate	4	0.79 ± 6.01	0.40 ± 0.00	2

### Antiviral ability of anionic compounds against influenza virus

**Fig. 1:**
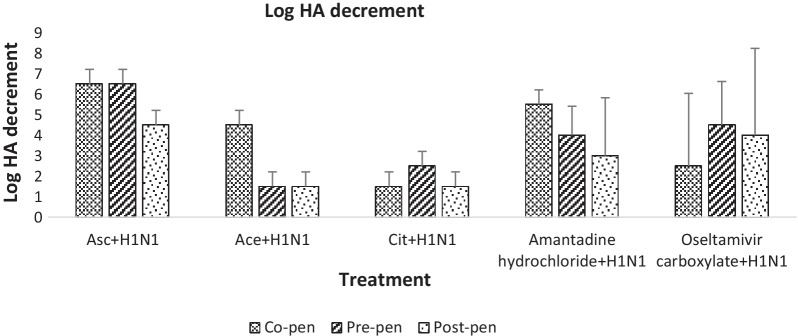
Log2 HA decrements. The log decrements were obtained from HA assay for the compounds combination treatments as compared to H1N1

The MTT test was performed as mentioned in methods section and the viabilities of mock-infected and infected cells were evaluated by determination of absorbance of formazan at 540 nm. Percentage of protection was calculated as follows: [(OD Sample-OD control positive)/(OD control negative − OD control positive)*100]. Data of cell viabilities and percentage of cell protection are shown in Tables 2 and 3, respectively. The antiviral capacity of the compounds was also measured by HA determining as shown in Additional file 1: Table S1. The log HA titer decrements are shown in Fig. 1, and Additional file 1: Table S2. As such, ascorbate, acetate, and citrate decreased the HA titer by 6.5, 4.5, and 1.5 logs in co-penetration; 6.5,1.5 and 2.5 logs in pre-penetration; and 4.5,1.5 and 1.5 logs in post-penetration experiments, respectively. Amantadine and oseltamivir were tested in parallel, with amantadine and oseltamivir showing the best response in co-penetration (5.5 ± 0.71 log decrement) and pre-penetration (4.5 ± 2.12 log decrement), respectively. Our desired compounds showed significant and even better results compared to the standard drugs. The estimated marginal means of cell viability, percentage of protection and log HA decrement after GLM analysis are shown in Additional file 1: Figure S1.

**Table 2:** Cell viabilities from MTT assay in combined treatments compared to H1N1

Compound	Cell viability (mean ± SD)		
	Co-pen	Pre-pen	Post-pen
Ascorbate + H1N1	2.97 ± 0.01**	1.82 ± 0.79**	3.69 ± 0.21**
Acetate + H1N1	1.11 ± 0.02**	1.08 ± 0.02**	0.82 ± 0.09**
Citrate + H1N1	1.34 ± 0.41**	1.03 ± 0.04**	0.92 ± 0.03**
Amantadine hydrochloride + H1N1	0.89 ± 0.03**	0.52 ± 0.04*	0.54 ± 0.05*
Oseltamivir carboxylate + H1N1	0.98 ± 0.00**	0.93 ± 0.00**	0.92 ± 0.03**
H1N1	0.23 ± 0.04	0.23 ± 0.04	0.23 ± 0.04

Data presented as mean ± SD are averages of 4 independent MTT assays

*Significantly and** highly significantly different from untreated sample (*P* ≤ 0.05 and *P* ≤ 0.01) were analyzed by SPSS, Tukey post-hoc test

**Table 3:** Percentage of cell protection against H1N1 in combination treatments

Sample	Percentage of protection (mean ± SD)		
	Co-pen	Pre-pen	Post-pen
Ascorbate + H1N1	505.12 ± 2.35	287.06 ± 150.79	642.65 ± 40.03
Acetate + H1N1	150.66 ± 3.05	145.35 ± 3.95	94.52 ± 17.04
Citrate + H1N1	193.77 ± 77.98	135.74 ± 7.25	115.40 ± 30.31
Amantadine hydrochloride + H1N1	57.70 ± 2.73	22.08 ± 4.11	35.68 ± 27.93
Oseltamivir carboxylate + H1N1	67.39 ± 0.36	62.95 ± 0.70	65.63 ± 8.01

The samples of co-penetration treatments were quantified by TCID_50_ evaluation to confirm the antiviral results.

### Cytokines profile analysis

To investigate the effect of viral infection and anionic compounds on the production of inflammatory cytokine such as IL-6, CCL2 and TNF-α as well as anti-inflammatory cytokine including IL-27, IFN-β1, ELISA tests were performed on the cell culture supernatants of co-penetration treatments which showed better results in previous steps. The cytokine protein levels in supernatants of MDCK cell culture at 72 h after exposure were calculated according to the reaction standard formula.

**Fig. 2:**
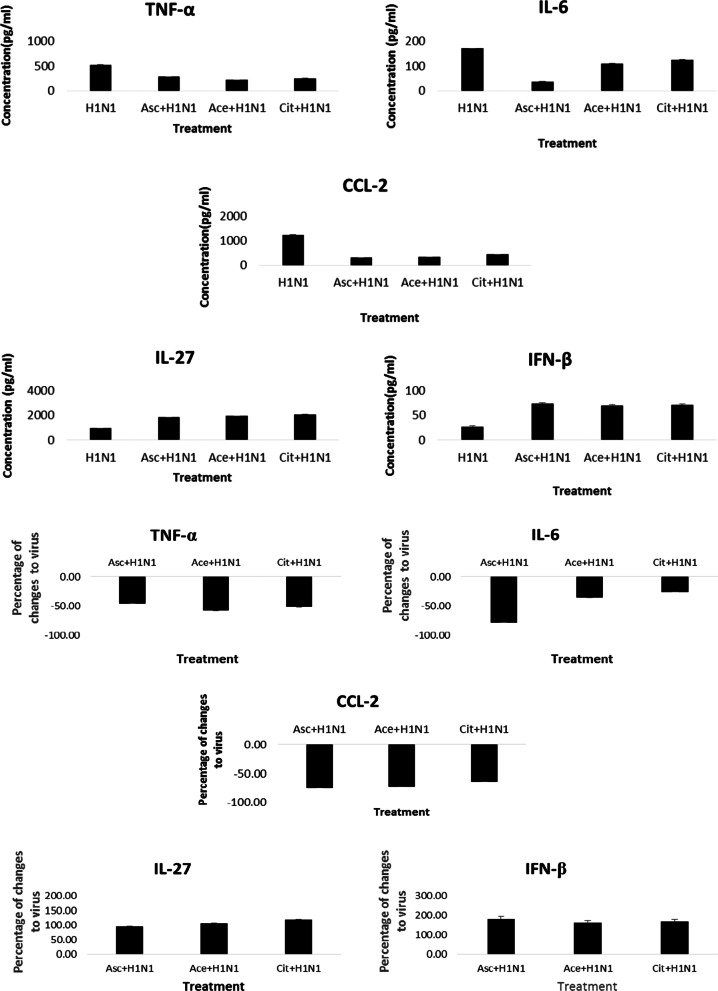
Concentrations and percentage of changes of cytokine proteins relative to H1N1 as determined by ELISA. The upper panel shows the concentrations of the cytokine proteins. The lower panel shows the percentage of changes of cytokine proteins relative to H1N1

To calculate the concentration of the cytokines in cell free supernatants, firstly, the standards optical densities were measured and the standard curves were plotted. The equations of standard curves were used to estimate protein concentration in the samples. The cytokine concentrations and percentage of changes are shown in Fig. 2, and Additional file 1: Table S3. Combination treatments significantly reduced pro-inflammatory and increased anti-inflammatory cytokines compared to the virus.

According to the results of TNF-α, the largest decrement was related to acetate combination treatment. In the case of IL-6 and CCL2 cytokines, the greatest decrease was related to the combination treatment of citrate (126.60 ± 0.17) and ascorbate (309.05 ± 1.86).

In the case of two anti-inflammatory cytokines IL-27 and IFN- β, the highest increase was observed in combination treatments of citrate (2055.00 ± 0.00) and ascorbate (73.75 ± 1.77) with the virus.

### Molecular data analysis

The one-step PCR for IAV M gene detection showed the Ct value of 12.76 ± 0.24 for H1N1 and for combination treatments of acetate and citrate with H1N1 showed 20.70 ± 0.30 and 22.45 ± 0.75, respectively. No Ct value was detected for ascorbate combination treatment.

**Fig. 3:**
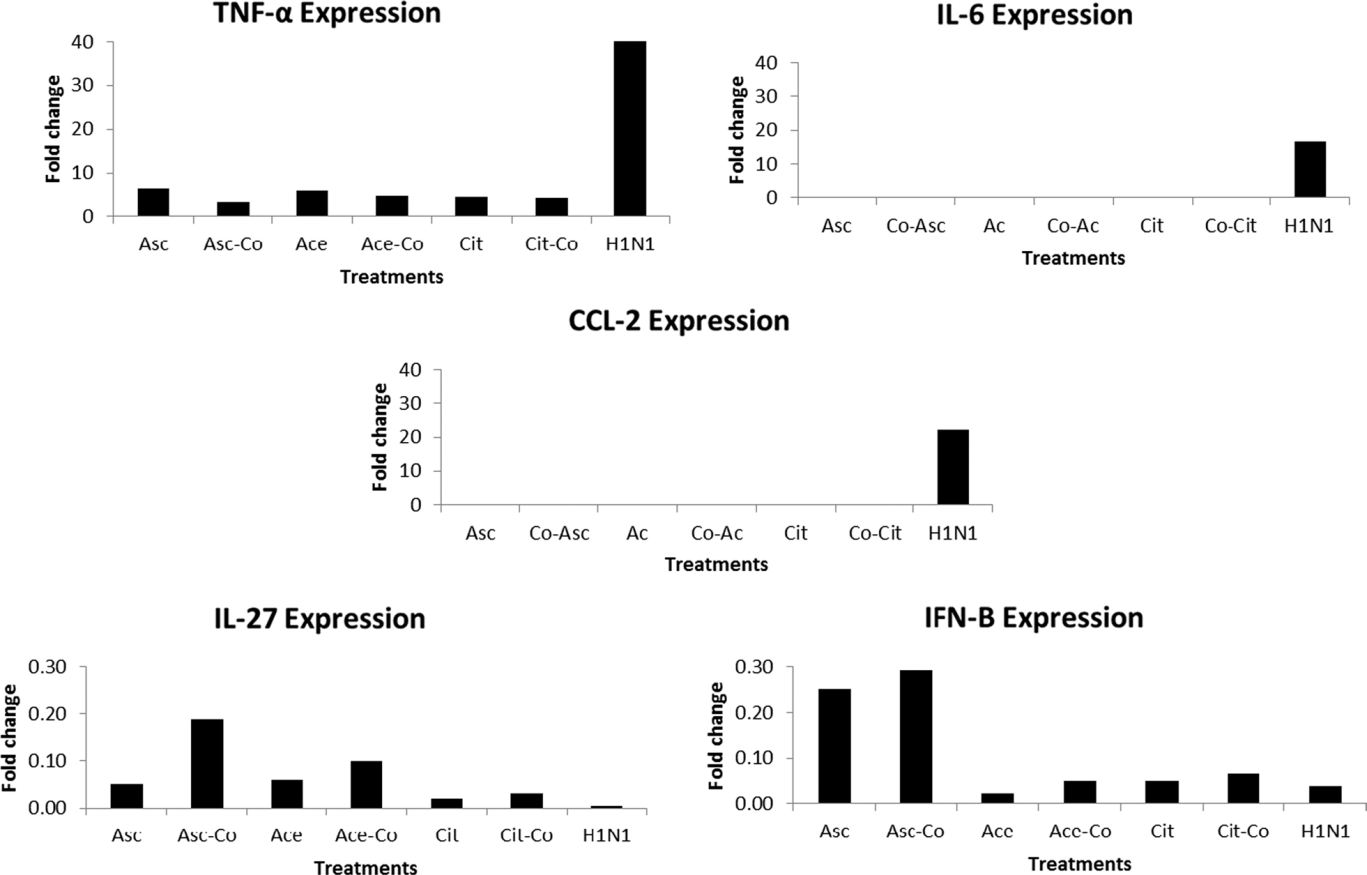
Relative expression analysis of the genes calculated as fold change compared to H1N1. Co: co-penetration

For the relative expression analysis of the cytokines genes, the Ct values of the genes were standardized by expression of the reference genes (average of the Ct values of two housekeeping genes). The relative expression of the genes was calculated as fold change compared to the positive control and data are shown in Fig. 3. H1N1 inoculation increased TNF-α expression to 191.00 fold but for the combination procedure, ascorbate, acetate and citrate decreased this ratio to 3.36, 4.83 and 4.26 fold, respectively. Accordingly, IL-6 expression increased to 16.622 fold in H1N1 inoculation but in the combination treatment, ascorbate, acetate and citrate decreased this cytokine expression ration to 0.00, 0.020 and 0.029 fold, respectively. The CCL2 expression in viral inoculation increased to 22.123 fold but in the combination treatment, ascorbate, acetate and citrate decreased this ratio to 0.165, 0.094 and 0.034 fold, respectively. With regard to anti-inflammatory cytokines; the level of IL-27 and IFN- β1, H1N1 reduced to 0.004 and 0.038 fold, respectively. In the combination experiments, ascorbate, acetate and citrate increased the IL-27 level to 0.188, 0.099 and 0.031 fold, and elevated the IFN- β1 level to 0.293, 0.049 and 0.065 fold, respectively, with no considerable change at the genomic level, except for ascorbate which affect IFN- β1 gene expression level.

## Discussion

In this study, we evaluated anti-influenza activity of three anionic compounds; ascorbate, acetate and citrate against influenza A virus PR/8/34 H1N1, looking at their capacity to decrease viral load while maintaining cell viability and affect the cytokine profile at genomic and protein levels.

One of the key agents of influenza pathogenesis is that it can cause uncontrolled cytokine production [1]. Corticosteroids have not been successful in controlling excessive inflammation [39]. There is enough information to support the idea that a combination of anti-inflammatory treatment for inflammation-associated lung injury with antiviral factors to control the diffusion of viruses would be more effective therapies for the infectious illnesses [40]. Over-the-counter drugs which are more accessible to the general public can be used as repurposing therapeutics especially at early stages of infection.

The main viral glycoprotein, Haemagglutinin, is engaged with the connection of flu viruses to their sialylated cell-surface receptors. This protein is pH sensitive and undergoes conformational changes in acidic conditions inside the cell, which helps virus propagation [41]. However, immature exposure of the virus to low pH in the extracellular environment might induce conformational substitutes to glycoprotein spikes on the virus surface and interfere with its binding to the cell that provides an attractive target for the antiviral evaluation [42]. Low pH aggregation of ribonucleocapsids has been observed before [43].

The effect of a natural citrus mixture containing citric acid has been assessed against influenza in vitro and in vivo. Using this mixture, the viral load in tracheal and lung tissue of 2 weeks old boilers infected with virus reduced significantly. This mixture decreased the clinical signs and mortality rate in treatment group. Besides, a considerable reduction was observed in pro inflammatory cytokines [44]. In a review study was mentioned that, itaconate produced by citrate ameliorated anti-inflammatory response. It was shown that citrate was needed for the immune cell activation and function [45]. In a study conducted at 50 years ago, low pH gel intranasal spray of Resiguard F (mixture of benzalkonium chloride, Triton X100, and citric acid) inactivated influenza virus in vitro and protected ferrets against influenza virus infection. It showed a remarkable virucidal effect on lipid-containing deoxyribonucleic and ribonucleic acid viruses, such as vaccinia virus, herpesvirus, and influenza virus [46]. In a research conducted by Kandi et al., they measured the inhibitory effect of G2 (a biocompatible anionic citrate-PEG-citrate dendrimer) on HIV infection. The dendrimer exhibited antiviral properties (ICC_50_: 0.4 mM) and minor toxicity (CC_50_: 0.6 mM) [47].

A derivative of acetate was studied in vitro and in vivo using mouse model of RSV infection. Pre-treatment of acetate on A549 cells resulted in reduced viral load and increased interferon stimulated genes. RSV-infected mice showed decreased load of virus after intranasal treatment with acetate. Also, treated mice recovered faster with a shorter duration of fever and increased expression of IFN-β in lung tissue [48].

Previously, a new function for the effects of vitamin C on influenza virus-induced pneumonia was shown in restraint-stressed mice. The results showed that restraint stress considerably enhanced the mortality and severity of respiratory illness in mice infected with A/FM/1/47(H1N1) and manifestation of the disease was attenuated by oral administration of vitamin C (125 and 250 mg/kg) [49]. Available studies on the beneficial effects of using ascorbic acid (vitamin C) during the course of IAV infection are based on the retrospective observational studies, which have shown that vitamin C reduces morbidity during influenza pandemics and it can decrease seasonal influenza outbreaks [50]. An examination showed that the antiviral capacity of dehydroascorbic acid was probably due to its binding to the virus surface or the molecules involved in viral replication and it may cause changes in the antigenicity of the hemagglutinin molecule of H3 influenza virus at acidic pH [51]. Pharmacological vitamin C as a pro-drug has been shown to eliminate or kill the influenza virus, possibly through generating steady-state concentrations of hydrogen peroxide (H_2_O_2_) in the extracellular fluid [52].

A study in 2015 evaluated mortality rate in mice model infected with Venezuelan encephalitis virus. In mice group that were treated with vitamin C, the mortality was half of the control group, with a marked decrease in viral titer [53]. A recent population study presented several evidence for the use of vitamin C and quercetin as synergistic prophylaxis and the early treatment in high-risk populations and as a treatment for respiratory tract infections, especially COVID-19 patients.

[54].With careful examination of our proposed compounds regarding effectiveness against influenza virus, the current study determined the effects of ascorbate, acetate and citrate in vitro to reduce the ability of IAV to proliferate.

The MTT and HA assays were conducted in different combination treatments to verify the effect of the compounds on the cell viability and viral load, respectively.

The anionic compounds were able to reduce the titer of influenza virus in all combination treatments. While all compounds were able to inhibit virus replication in vitro, ascorbate in particular had a significant effect on reducing virus titers with more than 6 logs HA decrement (Fig. 1). Given that these standard drugs had good efficacy against the virus, due to the reported drug resistance to them, the anionic compounds tested in this experiment could be favorable alternatives. The microscopic examination was in conformity with the cytotoxicity results of MTT assay that all CC_50_ and above concentrations were toxic against the cell viability and changed the cell morphology somewhat. The NCTC (200 mg/ml for ascorbate and 3 mg/ml for acetate and citrate) with no significant toxicity on the cell viability were selected for antiviral assay. Although, all the combination treatments of these concentrations displayed great efficiency, the best outcome was obtained at co-penetration treatments (Table 3), which was selected as a choice of combination method for the further experiments.

The effect of the compounds on H1N1 proliferation was also examined using molecular assay. Viral gene level in all treatments was measured by one-step quantitative real-time PCR comparing the Ct values of the combination treatments with H1N1, which clearly showed viral replication decrement in exposure to the compounds. The results indicated the inhibition of viral replication by all three compounds especially for ascorbate treatment. This outcome was in accordance with TCID_50_ results. In comparison with the virus (10^6.5^), ascorbate, acetate and citrate reduced the infectious dose of the virus by 6.5, 1.75, and 2.25 logs, respectively.

The pathogenesis of the influenza virus is a result of combination of host and virus elements. The virus particle induces replication of the virus in the target cell and additionally deceives the host immune system. It has been reported that the fatal consequence of influenza is eminently related to an enormous viral load along with high cytokine deregulation, which causes a cytokine storm or hypercytokinemia of both pro-and anti-inflammatory cytokines [55]. Hence, the innate immune system can affect the clinical manifestation and fatality following influenza virus infection by controlling inflammatory responses [56]. Hence, the compounds with immunomodulatory properties can also help this process.

In this study, TNF-α, IL-6 and CCL-2 were chosen from the list of pro-inflammatory cytokines and IL-27 and IFN-β were selected from the category of anti-inflammatory cytokines. These factors were tested at the genomic and protein levels. It was observed that our proposed compounds changed the state of cytokine production during the influenza infection.

For the relative expression evaluation of the cytokines genes, the ΔΔCt method was used and indicated that our proposed anionic compounds could reduce the H1N1 effect on the examined cytokines at the genomic level (Fig. 3).

ELISA also confirmed that infection by H1N1 causes high and low levels of target pro-inflammatory and anti-inflammatory cytokines expression, respectively in MDCK cells, while in all combined treatments of anionic compounds the responses were modulated in a good manner (Fig. 2, Additional file 1: Table S3).

One of the affected cytokines in influenza infection is TNF-α and it has been shown that the expression of TNF-α is induced [57]. This cytokine is effective in acute responses including inflammation, fever, and inhibition of viral replication [57, 58]. It can also activate NF-κB via TNF receptors, which mediate molecules involved in cell survival and inflammatory reactions [58].

Some reports have demonstrated that IL-6 plays a fundamental role in protection against influenza virus-induced lethal lung pathology [59, 60]. This cytokine is extremely correlated with fever during influenza disease [61], and strong up-regulation of this cytokine can cause high intensity of the infection [62].

One of the affected chemokines in flu infection is chemokine (C–C motif) ligand 2 (CCL-2). The role of this chemokine is the recruitment of the dendritic and macrophage cells to the infection site [63, 64]. Attraction of these inflammatory cells to the site could cause TNF-α manufacturing [64]; though, excessive recruitment of these cells would have lethal consequences [65]. Reduction in CCL-2 and trafficking of mononuclear cells to the infection site can efficiently ameliorate the lethal effects of influenza [66].

Ascorbate, acetate and citrate were able to significantly decrease the TNF-α, IL-6 and CCL-2 protein expression (Fig. 2, Additional file 1: Table S3). The results were in accordance with considerable decrements in their expression at the genomic level (Fig. 3). Therefore, the anionic compounds especially ascorbate, which is already used against inflammatory reactions with high capability to decrease the fever of flu, can control the extreme innate inflammatory reaction.

The anti-inflammatory cytokines studied in this research were IL-27 and IFN-β. IL-27 can augment the production of IL-10 by the antiviral CD4 + cytotoxic T lymphocytes (CTLs), which can efficiently moderate immune response irregulation [67]. IFN-β, which is a type I interferon that suppresses inflammation [68] can shift the cytokine networks in favor of anti-inflammatory responses [69].

The ascorbate, acetate and citrate could enhance the IL-27 protein level significantly compared to the H1N1. At the protein level, all compounds were able to significantly enhance the level of IFN-β in comparison with H1N1. This effect was in agreement with the expression study especially for ascorbate.

From the outcomes of the current study it is assumed that the proposed anionic compounds may have the capacity to inhibit virus by blocking the attachment of hemaglutinin to its receptor on the cell surface from the first step. In co-penetration treatment, the surface receptors of the virus are affected first. Since the co-penetration combination showed the lowest virus titer, it can be interpreted that the compounds can directly affect the virus surface receptors. However, the complementary experiments would help to prove this idea. The compounds may also indirectly control the virus outcome by affecting the cytokine profiles.

According to the references the relative safety of the compounds are determined by the selectivity index (SI). In general, an SI of 10 or greater is indicative of positive antiviral activity, although compounds with low SI are also taken into consideration. Natural compounds having confirmed SI values of less than 3 are also proper to be evaluated [35]. As also shown in other studies some compounds had low SI but good antiviral activity [70].

Given that the SI of all the compounds we studied was below 10, but they showed good antiviral activities, it can be said that this rule may not be always true and the compound should not be neglected. In this study, ascorbate with even low selectivity index value of 1.43 was the most effective compound. However, to apply it in clinical studies various criteria in addition to SI should be considered.

However, this study has potential limitations; it is possible to study different time points to evaluate the effectiveness of these compounds at different time intervals. According to the results, these compounds are also worth studying on cellular pathways such as apoptosis and autophagy.

## Conclusion

In conclusion, the proposed anionic compounds in this study especially ascorbate, are safe and effective against influenza virus. It is possible to improve the disordered function of immune system by modulating the inflammatory responses if proper treatment is applied. We proposed for the first time the potential capability of these compounds to ameliorate effectively the consequences of the influenza disease by inhibition of the virus attachment to the HA receptors on the cell and indirectly by affecting cytokine profile. As these compounds, showed the best activity in co-penetration treatments, the proposed therapeutic approach could be taking the treatment with the onset of the first symptoms of the viral disease that can prevent the binding and proliferation of more viruses.

Further evaluation of the mechanism of action of these compounds would shed light on proper ways to release the burden caused by not only influenza virus but also other respiratory viruses that may have similar pathogenesis.

## Supplementary Information

**Additional file 1. Supplementary Figure 1**. Estimated marginal means of (a) cell viability, (b) percentage of protection, (c) log HA decrement analyzed by GLM.** Supplementary Table 1**. Raw data of the HA test results.** Supplementary Table 2**. Log2 HA decrement assessment in different combination. ** Supplementary Table 3**. Concentration and percentage of changes of cytokine proteins relative to H1N1 as determined by ELISA.

## Data Availability

All data are available in case of need.

## Abbreviations

AA: Ascorbic acid
ACTB: Actin beta
CC_50_: Cytotoxicity concentration 50%
CCL2: C–C motif chemokine ligand 2
DMED: Dulbecco’s modified Eagle’s medium
EV71: Enterovirus 71
ELISA: Enzyme-linked immunosorbent assay
FAC: Ferric ammonium citrate
FBS: Fetal bovine serum
GAPDH: Glyceraldehyde 3-phosphate dehydrogenase
GusB: Glucuronidase beta
GLM: General linear model
HA: Haemagglutination assay
HIV: Human immunodeficiency virus
HSV-2: Herpes simplex virus 2
IAV: Influenza A virus
IFN-γ: Interferon-γ
IL: Interleukin
MDCK: Madin-Darby Canine Kidney
MTT: [3-(4,5-Dimethyl-2-thiazolyl)-2,5-diphenyl-2H-tetrazolium bromide]
NAC: N-acetyl cysteine
NCTC: Non-cytotoxic concentrations
PBS: Phosphate-buffered saline
SI: Selectivity index
TCID_50_: Median tissue culture infectious dose
TNF-α: Tumor necrosis factor-α
