# Correction to: In vitro anti-influenza assessment of anionic compounds ascorbate, acetate and citrate

**DOI:** 10.1186/s12985-022-01832-z

**Published:** 2022-06-21

**Authors:** Hadiseh Shokouhi Targhi, Parvaneh Mehrbod, Fatemeh Fotouhi, Mehriar Amininasab

**Affiliations:** 1 Department of Cell and Molecular Biology, Kish International Campus, University of Tehran, Kish Island, Iran; 2 Influenza and Respiratory Viruses Department, Pasteur Institute of Iran, Tehran, Iran; 3 Department of Cell and Molecular Biology, School of Biology, College of Science, University of Tehran, Tehran, Iran

## Correction to: Virology Journal (2022) 19:88 https://doi.org/10.1186/s12985-022-01823-0

Following publication of the original article [1], the authors would like to correct the affiliation of author Hadiseh Shokouhi Targhi and author Mehriar Amininasab. Also they corrected the Author contributions.

section.

The corrected Author contributions should read:

The idea and design of project: MA; designed the experiments: HST, FF, PM; performed the experiments: HST; Analyzed data: HST, FF, PM; Wrote the paper: HST; Comprehensive reading and editing of the manuscript: MA, FF, PM.

The authors affiliation has been updated above and the original article [1] has been corrected.
