# Supplementary material for: In vitro anti-influenza assessment of anionic compounds ascorbate, acetate and citrate
Source: Virol J. 2022 May 23;19:88. doi: 10.1186/s12985-022-01823-0 (PMC9125540; doi:10.1186/s12985-022-01823-0)
Supplement: Supplementary file 1 — Additional file 1. Supplementary Figure 1. Estimated marginal means of (a) cell viability, (b) percentage of protection, (c) log HA decrement analyzed by GLM. Supplementary Table 1. Raw data of the HA test results. Supplementary Table 2. Log2 HA decrement assessment in different combination. Supplementary Table 3. Concentration and percentage of changes of cytokine proteins relative to H1N1 as determined by ELISA. [file 12985_2022_1823_MOESM1_ESM.docx]

**Supplementary Figure 1.** Estimated marginal means of (a) cell viability, (b) percentage of protection, (c) log HA decrement analyzed by GLM.

Line 1: Co-penetration, Line 2: Pre-penetration, Line 3: Post-penetration

X-axis: 1.00: Ascorbate, 2.00: Acetate, 3.00 Citrate, 4.00: Amantadine, 5.00: Oseltamivir, 6.00: H1N1. Y-axis title: (Estimated marginal means)

**Supplementary Figure 1.**


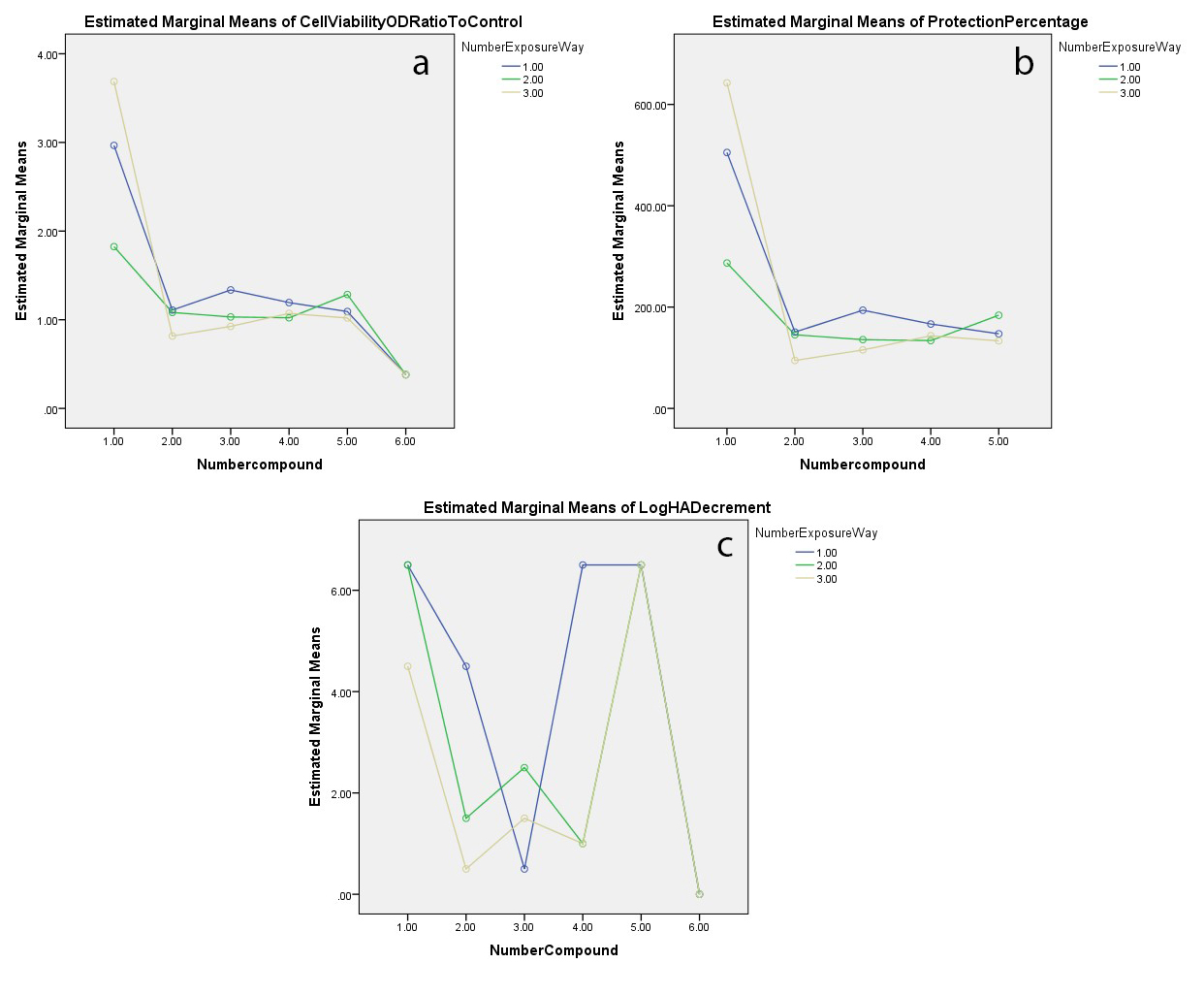


**Supplementary Table 1.** Raw data of the HA test results

| HA titer | | | | |
| --- | --- | --- | --- | --- |
| Treatment | **Combination treatments** | | | |
|  | **co** | **pre** | **post** | **H1N1** |
| Ascorbate+H1N1 | 0 | 0 | 4 | 64 |
|  | 0 | 0 | 4 | 128 |
| Acetate+H1N1 | 4 | 32 | 32 | 64 |
|  | 4 | 32 | 32 | 128 |
| Citrate+H1N1 | 32 | 16 | 32 | 64 |
|  | 32 | 16 | 32 | 128 |
| Amantadine+H1N1 | 0 | 8 | 32 | 64 |
|  | 4 | 4 | 4 | 128 |
| Oseltamivir+H1N1 | 64 | 8 | 32 | 64 |
|  | 4 | 2 | 0 | 128 |

**Supplementary Table 2.** Log2 HA decrement assessment in different combination treatments compared to H1N1.

| **Sample** | **Log2 HA decrement**  **(mean ± SD)** | | |
| --- | --- | --- | --- |
|  | **Co-pen** | **Pre-pen** | **Post-pen** |
| **Asc+H1N1** | 6.50 ± 0.71 | 6.50 ± 0.71 | 4.50 ± 0.71 |
| **Ace+H1N1** | 4.50 ± 0.71 | 1.50 ± 0.71 | 1.50 ± 0.71 |
| **Cit+H1N1** | 1.50 ± 0.71 | 2.50 ± 0.71 | 1.50 ± 0.71 |
| **Amantadine hydrochloride+H1N1** | 5.50 ± 0.71 | 4.00 ± 1.41 | 3.00 ± 2.83 |
| **Oseltamivir carboxylate+H1N1** | 2.50 ± 3.54 | 4.50 ± 2.12 | 4.00 ± 4.24 |

Supplementary Table 3. Concentration and percentage of changes of cytokine proteins relative to H1N1 as determined by ELISA.

| **cytokine protein concentration (pg/ml) (mean ± SD)** | | | | | |
| --- | --- | --- | --- | --- | --- |
| **Treatment** | **TNF-α** | **IL-6** | **CCL2** | **IL-27** | **IFN- β** |
| **Asc+H1N1** | 284.25 ± 0.32** | 38.05 ± 0.17** | 309.05 ± 1.86** | 1833.33 ± 2.36** | 73.75 ± 1.77** |
| **Ace+H1N1** | 222.20 ± 0.64** | 110.64 ± 0.08** | 338.26 ± 0.00** | 1938.33 ± 0.00** | 68.96 ± 2.06** |
| **Cit+H1N1** | 253.45 ± 0.48** | 126.60 ± 0.17** | 446.95 ± 0.37** | 2055.00 ± 0.00** | 70.62 ± 2.06** |
| **H1N1** | 523.00 ± 2.41 | 171.78 ± 0.17 | 1243.79 ± 5.58 | 941.67 ± 4.71 | 26.25 ± 1.77 |
| **percentage of change of cytokine protein relative to H1N1 (mean ± SD)** | | | | | |
| **Treatment** | **TNF-α** | **IL-6** | **CCL2** | **IL-27** | **IFN- β** |
| **Asc+H1N1** | -45.65 ± 0.19 | -23.85 ± 0.02 | -75.15 ± 0.04 | 94.69 ± 1.22 | 181.36 ± 12.21 |
| **Ace+H1N1** | -57.51 ± 0.32 | -29.70 ± 0.08 | -72.80 ± 0.12 | 105.84 ± 1.03 | 163.03 ± 9.86 |
| **Cit+H1N1** | -51.54 ± 0.32 | -51.54 ± 0.32 | -64.06 ± 0.13 | 118.23 ± 1.09 | 169.39 ± 10.28 |

Data are expressed as pg/ml (N=2) for 72 h incubation time. **: highly significantly (*P*<0.01) different from H1N1-inoculated sample.
